# Editorial: Automatic methods for multiple sclerosis new lesions detection and segmentation

**DOI:** 10.3389/fnins.2023.1176625

**Published:** 2023-03-14

**Authors:** Olivier Commowick, Benoît Combès, Frédéric Cervenansky, Michel Dojat

**Affiliations:** ^1^Empenn INSERM U1228, CNRS UMR6074, Inria, University of Rennes I, Rennes, France; ^2^Univ Lyon, INSA-Lyon, Université Claude Bernard Lyon 1, UJM-Saint Etienne, CNRS, Inserm, CREATIS UMR 5220, U1206, Lyon, France; ^3^Univ Grenoble Alpes, Inserm, U1216, CHU Grenoble Alpes, Grenoble Institut Neurosciences, GIN, Grenoble, France

**Keywords:** neurodegenerative diseases, MRI, image processing, deep learning, neurology - clinical

Multiple Sclerosis (MS) is a chronic inflammatory disease of the central nervous system (CNS) affecting more than half a million persons in Europe, with a prevalence rate of 83 per 100,000 with higher rates in northern countries and a female/male ratio around 2.0 (Pugliatti et al., 2006). Today, conventional MR imaging (MRI) is widely used for the patient follow-up, the monitoring of the therapy effects, and more generally in a perspective of personalized medicine, for the understanding of the individual MS progression (Thompson et al., 2018). One of the major challenges in using MRI for MS is the segmentation of lesions whose number, location and appearance at a given time point, are crucial indicators for diagnostic and to tailor treatment to the specific individual disease's evolution.

To cope with inter- and intra-observer variability and reduce the burden and complexity of lesions identification for clinicians, a large number of techniques have been proposed in the literature for the automatic segmentation of MS lesions (see Garcia-Lorenzo et al., 2013; Valverde et al., 2017; Danelakis et al., 2018 for reviews). Several challenges have been proposed to evaluate the performances of these methods (e.g., Carass et al., 2017; Commowick et al., 2021 to cite the most recent ones). Moreover, recently Bonacchi et al. (2022) proposed an overview of Artificial Intelligence applications for MS clinical practice.

A growing literature focuses on the delineation of new MS lesions on T2/FLAIR occurring between two consecutive exams. Detecting the apparition of new MS lesions is of central interest in clinical practice. Indeed, while the palette of Disease Modifying Drugs (DMDs) approved for MS has presently an unknown impact on the compartmentalized neurodegenerative process within the CNS, they aim to substantially reduce, or even stop, the accumulation of new lesions. Consequently, the assessment of such an accumulation allows the clinician to monitor the efficiency of a given DMD on each patient it follows, and therefore to consider a change of treatment in case of insufficient efficiency. Moreover, there is a direct link between accumulation of new lesions and increasing handicap (Sormani et al., 2013). Automating the detection of these new lesions or helping clinicians to identify them would therefore be a major advance for evaluating the patient disease progression and response to treatment.

In 2021, we launched a MICCAI challenge, MSSEG-II (see https://www.ofsep.org/fr/etudes/msseg-ii-challenge-miccai-2021), to compare automated solutions for this specific task i.e., the detection of new lesions appearing at the second time point of two T2/FLAIR images of the patient. For that purpose, we used a large database: 100 patients, each with two time points, the time between the two time points varying between 1 and 3 years. Data were extracted from the national OFSEP cohort (Vukusic et al., 2020), the national French MS registry (https://clinicaltrials.gov/ct2/show/NCT03603457), with 3D FLAIR images from different centers and scanners (15 different scanners in total) using the OFSEP specific protocol (Cotton et al., 2015; Brisset et al., 2020). Only 3D FLAIR images—that is the mostly used clinical sequence for MS brain—were considered. As in our previous challenge (Commowick et al., 2021), the evaluation of solutions was performed on the dedicated FLI-IAM infrastructure (https://www.francelifeimaging.fr/en/about/noeuds/iam/), which comprises Shanoir, a web-oriented solution for imaging data storage and sharing for preclinical and clinical research studies (Barillot et al., 2016; Kain et al., 2020); and the VIP platform (Glatard et al., 2013) for the execution of the corresponding docker of each image processing algorithm/pipeline on EGI infrastructures (https://www.egi.eu/). The use of FLI-IAM allows to automate the competition's process through a sustainable framework and remove the potential biases (e.g., challengers manually optimizing their parameters for each provided case). The ground truth was defined based on the manual delineation, using ITK Snap, of the 100 cases by four neuroradiologists with an MS expertise. Then, a consensus was formed in two steps: a senior expert neuroradiologist examined and confirmed (or declined) disputed lesions among the experts; then a fusion using the STAPLE (Warfield et al., 2004) algorithm was performed. This consensus was then the reference for the evaluation procedure. Forty cases were provided to challengers (e.g., for algorithm training) and 60 cases for algorithm testing. The manual segmentations were provided with the former and unknown to the challengers for the latter.

The present RT gathers 10 papers about solutions for the automatized detection of new lesions in MS subsequent images. All but one (Dufresne et al.) competed during MSSEG-II challenge and were executed on FLI-IAM infrastructure. They are based on a deep learning approach, the U-net architecture (Ronneberger et al., 2015) with its 2D or 3D versions. We may distinguish two classes of approaches, ones that use exclusively the examples provided by the Miccai challenge organizers and those which introduce additional real (Hitziger et al.) or synthetic (Andresen et al.; Kamraoui et al.; Valencia et al.) datasets. Finally, joint modeling, mixing both a registration and a segmentation task, have been investigated (Andresen et al.; Dufresne et al.; Salem et al.).

Then, Hitziger et al. train a 2D U-net with residual units with axial, coronal and sagittal slices. The corresponding slices from the two time-point volumes are paired and introduced to the system as a two-channel input. The predictions from each orientation are then merged with different strategies. The best performances are obtained for the unanimous voting strategy where lesions are confirmed in each orientation. The gain in performance by introducing additional datasets (25 supplementary patients to the initial 40 patients training set) seems weak.

In the same line, Sarica and Seker propose a 2D U-net solution where the standard plain blocks are replaced by residual units and attention gates are introduced to, respectively, enhance the model performances and focalize on new MS lesions on each 2D slice. A majority voting generates the final 3D binary output.

Similarly, Ashtari et al. introduce residual units, this time in a 3D U-net version and data augmentation methods to improve robustness and generalizability of the obtained model.

Basaran et al. consider the recent 3D U-Net version (“No-NewU-Net”) combined with several image preprocessing step brain extraction, bias correction, registration and multiple data augmentation methods.

To overcome the difficulty of a supervised training based on scarce new lesion annotated examples, Kamraoui et al. interestingly propose to first pretrain a 3D U-Net on a large one time-point MS dataset (transfer learning), second to pretrain the model used for time-points by introducing realistic synthetic data, and finally to fine-tune the obtained network with the real two time-points data as provided by MSSEG-II.

To tackle class imbalance between voxels belonging to new lesions or not, Schmidt-Mengin et al. introduce a two-stage training strategy to iteratively define a fixed number of patches (30%) containing lesions. This “online hard example mining” strategy is implemented with two 3D U-Nets applied patch-wise in cascade. Such a strategy, applied for the first time on 3D brain scans, seems to emphasize false positive rate.

Instead of using a unique intensity-based approach, Andresen et al., Salem et al., and Dufresne et al. propose to consider a deformation-based approach. Maps of non-corresponding regions between subsequent images are generated during the registration process. In Andresen et al. such maps are then used by a fully convolutional network to segment new lesions that occur across time. Offset maps with baseline allow exploring morphology appearance of new lesions. New lesions are rare and similarly to the previous paper (Kamraoui et al.) the authors insert synthetic lesions during the network training. In Salem et al. the authors introduce a cascade of two 3D U-net patch-wise fully convolutional neural networks. The first registration network learns the deformation field to register the individual sequence of FLAIR images, while the second performs new lesions segmentation. The latter is fed by registered FLAIR images and the deformation maps. Indeed, the first network allows to filter the majority of non-lesion voxels and reveals the possible new lesion candidates, while the second refine the detection in reducing misclassified voxels. The simultaneous training of registration and segmentation modules improves the performances compared to a sequential learning. Valencia et al. propose to improve the previous results in adding synthetic images. The hypothesis is that the introduction of T1-weighted images (T1w), artificially generated, in addition to the FLAIR images improves new MS lesions detection. They use a generative adversarial network (GAN) with an additional MS FLAIR dataset (136 cases) in order to generate T1w corresponding images. The trained GAN is then used to generate the T1w corresponding to the provided MSSEG-II FLAIR images. They show an improvement of the sensitivity performance compared to the only use of FLAIR images.

Finally, in Dufresne et al., a different deformation-based approach is proposed where deformable registration and local intensity change detection are jointly estimated as a unified optimization problem solving. The joint method is evaluated on synthetic and real MS datasets and compared to the sequential version, where registration and change detection are performed successively, to demonstrate the performance improvement obtained by the former. Such an optimization approach cannot discriminate between new lesions from evolving lesions. It is interesting to note that this is the only non-Deep Learning-based method presented in this RT.

In Table 1, we provide several indexes for the readers in order to have a flavor of the current performances reached by the different solutions described in this RT compared to human experts.

**Table 1:** Averaged (patient-wise) score for the four experts.

**New lesion cases (*****n*** = **32)**									**No new lesion (*****n*** = **28)**	
	**DSC**	**F1**	**Sensitivity**	**Specificity**	**PPV**	η**TP**	η**FP**	η**FN**	η**FP**	η**FP**
Expert 1	0.629	0.709	0.650	1.000	0.707	6.063	1.281	1.094	0.036	1.453
Expert 2	0.597	0.601	0.526	1.000	0.813	4.500	0.844	2.375	0.000	0.000
Expert 3	0.535	0.637	0.580	1.000	0.760	4.313	1.094	2.500	0.107	3.981
Expert 4	0.459	0.519	0.407	1.000	0.801	4.469	0.594	2.375	0.036	0.623

DSC, dice score; PPV, positive predictive value; ηTP, mean number of true positives; ηFP, mean number of false positive; ηFN, mean number of false negative. The provided sensitivity, precision, and PPV are computed at the voxel scale.

To conclude, MS new lesions detection and segmentation remain very difficult tasks. Presently, automatic methods can be more sensitive for detecting new lesions, but produce more false positive compare to manual delineation by experts. Thus, in spite of slight persistent differences, performances between automatic solutions and human experts are closer than in the previous challenge (see Commowick et al., 2021). However, in order to be used in clinical routine, several steps need to be completed, such as the integration of computerized solutions in the hospital information flow and the quantification of the uncertainty associated to the automatic lesion detection, in place of the standard binary output, to leverage the clinician's work for obvious lesion and requiring his/her expertise only for difficult cases (Lambert et al., 2022). This will lead to the design of a new family of computerized medical assistants for care improvement.

Data from the MSSEG challenges are available here https://shanoir.irisa.fr/shanoir-ng/welcome and can be used to evaluate new solutions.

## Author contributions

All authors listed have made a substantial, direct, intellectual contribution to the work. BC, FC, and MD approved it for publication.

## Dedication

This editorial is dedicated to OC, our young and talented colleague who prematurely passed away in December 2022.
